# Managing Masculinity When Growing up With a Violent Father: A Qualitative Study of Boys’ Experiences

**DOI:** 10.1177/10778012241303462

**Published:** 2025-01-17

**Authors:** Jane E. M. Callaghan, Lisa C. Fellin, Stavroula Mavrou, Joanne H. Alexander, Vasiliki Deliyianni-Kouimtzis, Judith Sixsmith

**Affiliations:** 1 Centre for Child Wellbeing and Protection, 7622 University of Stirling, Stirling, UK; 2 Department of Human and Social Sciences, 18953 University of Bergamo, Bergamo, Italy; 3 School of Psychology, 37782 Aristotle University of Thessaloniki, Thessaloniki, Greece; 4 Faculty of Social Sciences, 7622 University of Stirling, Stirling, UK; 5 3042 University of Dundee, Dundee, UK

**Keywords:** boys, fathers, intimate partner violence, masculinity, qualitative research

## Abstract

A limited qualitative literature explores children's lived experiences of violence; boys’ relationships with perpetrator fathers remain largely unexplored. Drawing on interviews with 31 boys, this paper explores the accounts of their relationships with their perpetrator fathers, focusing particularly on the implications of boys’ understanding of these relationships for their sense of burgeoning masculinity. Three themes are considered: in (a) relational ambiguity; (b) performing masculinities, managing violence; and (c) envisioning alternative futures and re-visioning the past. Our findings highlight the importance of interventions for boys that facilitate the expression of their often complex and ambivalent feelings and fears about their father's violence, and what it means for them and their future.

## The Absent Trace: Fathers in Domestic Abuse Literature

To survive and make sense of a complex and fraught relational context, children who experience domestic abuse^1^ must negotiate family relationships characterized by aggression, coercion, and victimization. Although most research on children's experiences presumes a male perpetrator and female victim, there is little discussion of men *as fathers* in this research (Arnull & Stewart, 2021; Callaghan, 2024; Lapierre, 2008). Male violence is typically measured by proxy, based on the woman victim's report. When parenting is addressed in this research, it is almost exclusively the adult victim's parental functioning, mental health, and wellbeing that are assessed (Callaghan, 2015). Men are therefore largely invisible as *fathers* in literature on domestic abuse. This invisibility of men as domestic abusers is also evident in social work and other professional practice with women and children impacted by domestic abuse (Humphreys et al., 2018; Mandel, 2010; Witt & Diaz, 2019) and contributes directly to the problematization of women as mothers when domestic abuse occurs (Fauci & Goodman, 2020; Wild, 2022).

In contrast, mothers’ parenting and mental health are routinely measured and correlated with children's, and women are positioned in this literature as the main mediators of children's positive or negative psychosocial outcomes (Callaghan, 2015, 2023; Featherstone & Peckover, 2007; Øverlien, 2011). The violence committed by men, measured indirectly, becomes indistinct, reduced to the reported rate and intensity of the act of violence, but abstracted from the relational context of the family. Reduced to a single variable (level of violence), violence and abusive behaviors can be more easily discounted, rendered unimportant to the impact of domestic abuse on children's wellbeing (Schechter et al., 2011). The mother's mental health, her parenting style, her addictions, and her emotional availability are all intensely scrutinized (Holmes, 2013; Levendosky et al., 2006; Milford & Oates, 2009), and women's capacity to cope with their own distress, and to mother “effectively” are positioned as the key factor in children's recovery from domestic abuse (Schechter et al., 2007; Whitaker et al., 2006). For example, in a recent systematic review of resilience in children impacted by domestic violence and abuse (Fogarty et al., 2019), of the 15 studies included, only two examined individual child factors (Bowen, 2017; Martinez-Torteya et al., 2009), two assessed the perpetrator parent's behaviors through maternal report, while nine reported on maternal mental health, and 12 assessed aspects of maternal parenting and attachment. A similar pattern is evident in Cameranesi et al.'s (2020) scoping review. Both studies concluded that maternal mental health and parenting predicted children's outcomes.

While mothering is extensively studied, and often pathologized (Morgan & Coombes, 2016), the father's relational role and mental health functioning are obscured. When men and fathers are considered, the male abuser is predominantly described in reductionist and monolithic terms as *just* violent, without consideration of any other kind of agency, relationality, or personhood. In the context of domestic abuse support services, the representation of the abuser as a violent object is widespread. As Featherstone and Peckover (2007) have suggested, this enables “the discursive removal of violent men from the category of father” (p. 181), producing a range of challenges in supporting these families (Birchall & Choudhry, 2022).

Some qualitative studies have considered children's perceptions and experiences of their violent fathers. Lapierre et al. (2022) conducted interviews and focus groups with 59 children and young people, exploring their relationships with their fathers. They found that children expressed predominantly negative views about their fathers, positioning them as violent and manipulative, and expressing relief that they had limited or no contact with them. However, in other qualitative research, these experiences are often described as contradictory and complex (DeBoard-Lucas & Grych, 2011; Staf & Almqvist, 2015). Many children represent their father in polarized ways, as either all good or all bad, or manage competing experiences of their fathers, including positive and negative feelings (Cater, 2007; Mullender et al., 2003) and disjunctive images (Staf & Almqvist, 2015). Contact with fathers post-separation is a particularly fraught experience for children, with many reporting that they still felt fear of their fathers (Lapierre et al., 2022; Øverlien, 2013), some wanting no contact, while others describe missing him and desiring some contact (Henze-Pedersen, 2021; Mullender et al., 2003). Some children reported manipulation by fathers post-separation, or felt drawn into a sense of constant conflict in which their emotions and self-expression had to be constantly monitored and managed (Holt, 2015; Staf & Almqvist, 2015). The balance of literature suggests that children's experiences of their relationship with their fathers is located in a complex set of interrelationships between mothers, fathers, and children, pointing to the relational complexity which children negotiate with regard to their location within family life (Cater & Øverlien, 2014; DeBoard-Lucas & Grych, 2011; Henze-Pedersen, 2021; Øverlien, 2013).

The limited engagement with fathers in the academic and practitioner literature results in few resources to help children make sense of their relationships with violent fathers or stepfathers.^2^ This is complexified by a practitioner landscape in which many domestic abuse specialists and social workers lack training in engaging with perpetrators, particularly in uncovering and managing their often manipulative and hidden patterns of coercive control (Stanley et al., 2019; Stanley & Humpreys, 2017).

We need theoretical accounts and practice that enable children to work through their ambivalence about their fathers, and to explore its meaning in how they make sense of themselves and their futures (Fellin et al., 2019). One concept commonly used to make sense of the link between domestic abuse, parenting, and children's outcomes is intergenerational transmission—the idea particularly that children who grow up in households where domestic violence and abuse occur are more likely to be involved in abusive relationships themselves, as either victims or perpetrators (Ehrensaft & Langhinrichsen-Rohling, 2022).

Intergenerational transmission was initially framed through a lens of social learning theory, with children seen as picking up abusive behaviors through observation and imitation in the home environment (O’Leary, 1988). However, more recent research focuses on neurodevelopmental pathways, suggesting that violence and abuse in the home disrupts neurophysiological development involved in the human stress response, and that this disrupts emotional regulation, and produces an enduring vulnerability to social and emotional challenges (Margolin et al., 2016). (Though, as Cherry and Gerstein (2021) have argued, most literature on the role of parents in emotion regulation skill development has focused on maternal emotion socialization, neglecting fathers’ contributions to children's emotional development.) These concepts have been widely advanced through, for example, the popularization of trauma-informed and adverse childhood effect-informed approaches to understanding violence in home. In general, boys and men are far more likely to perpetrate violence than women and girls, an association that has long been explained with reference to the construct of masculinity (see, e.g., Flood et al., 2023; Jewkes et al., 2015). While boys who experience violence and abuse in childhood are certainly not “doomed to repeat” that cycle (Alexander, 2022; Radford et al., 2019), there is evidence that boys who experience domestic abuse in childhood have a slightly increased risk of involvement in future domestic violence and abuse (Ehrensaft & Langhinrichsen-Rohling, 2022). However, the notion of “transmission” is necessarily mechanistic and reductionist. What is missing in this kind of account is an understanding of how boys experience and make sense of the domestic abuse that they live with. The evidence that far more boys do not go on to be abusive suggests the need to understand in more detail the relational terrain that boys navigate as they grow up with violent and abusive fathers. It therefore is particularly important to untangle some of the ambivalence and ambiguity inherent in their relationships with their fathers, and their associated understanding and experience of masculinity (Fellin et al., 2019). A more nuanced understanding of the relationships between boys and their perpetrator fathers/stepfathers is needed to help them make sense of their relational context (Cooper & Vetere, 2008; Fellin et al., 2019). This analysis must extend beyond simple concepts like “role models” and “bad fathers” to understand the complexity of this relational terrain.

Such an endeavor must also be placed within the context of a court system that prioritizes fathers’ access to children regardless of their status of perpetrators of domestic violence and abuse (Lapierre et al., 2022; Morrison et al., 2020). There is a widespread failure in the justice system internationally to recognize that domestic abuse is not constrained to the intimate dyad, and that it extends beyond isolated physical incidents to impact the entire relational terrain of the family (Callaghan et al., 2017, 2018, 2019; Katz, 2016; Swanston et al., 2014).

In this article, we extend the small body of qualitative work on children's relationships with their fathers, with specific reference to the experiences of boys. By exploring the often fraught father–son relationship we consider the implications of this relational experience for boys’ sense of identity and their understanding of themselves as relational beings. In this article, we explore the intertwining of boys’ relational experiences of their fathers or stepfathers, and their understandings of masculinities, growing up in a family where masculinity has been expressed in problematic, violent, and abusive ways. Our aim is to explore how boys experience and understand their relationships with their fathers or stepfathers, and how they both take up and resist dominant constructions of masculinity, to constitute a sense of themselves as boys and men.

### Understanding Masculinities

To make sense of boys’ understanding and experiences of their relationships with their violent and abusive fathers, we draw on a theoretical understanding of masculinities as socially constructed and performative (West & Zimmerman, 2009), moving beyond a concern with violent male *role models*, to consider how masculinity is negotiated and accomplished by boys in families where there is a violent man. To do this we explore the symbolic and cultural resources available to boys to make sense of themselves as male. Masculinity is not understood as a unitary phenomenon, but as contradictory and complex (Morris & Ratajczak, 2019). While we maintain that masculine identities are formed in reference to hegemonic masculinity (i.e., forms of masculinity associated with power, physical strength, and dominance) (Channon & Matthews, 2015; Connell & Messerschmidt, 2005), this functions as one cultural resource men and boys use in constituting and understanding themselves as masculine subjects. We are interested here, not in what masculinity *is* but rather in the cultural resources available to boys to make sense of what it means to be male, male violence, and their own masculinity.

Recent public debates have focused on constructs like “fragile” (Pascoe, 2007; Pascoe & Hollander, 2016), toxic (DeBoise, 2019), or “threatened” masculinity (Cohn et al., 2009). Social psychologists have documented how perceived threats to hegemonic masculinity might result in male anger and violence toward women (see, e.g., Dahl et al., 2015). Dimuccio and Knowles (2020) suggest that hegemonic “manhood” bestows male privilege, but that it also demands that men demonstrate their masculinity and defend their claim to the status it bestows. They suggest that for some men, this produces anxiety, and compensatory macho attitudes and behaviors. They define fragile masculinity as “the anxiety that stems from manhood's precariousness and the behavioral consequences of this anxiety” (DiMuccio & Knowles, 2020, p. 25). Similarly, male aggression is sometimes theorized through the concept of “hypermasculinity,” another construct that frames aggressive posturing, toughness, violence, and misogyny as a brittle response to male shame and inadequacy (Archer & Yamashita, 2003; Ray & Parkhill, 2021). This notion of aggressive forms of masculinity as “fragile” emphasizes the performative nature of masculinist posturing, and suggests that men who use violence are expressing a weakness in their masculine identity, rather than a sense of security and personal strength. These concepts have not been extensively deployed in feminist or other theorizing about domestic violence and abuse, but are very visible in popular representations and debates around male violence (e.g., D’Avanzato et al., 2022; Jones et al., 2022) as are other highly individualizing popularized conceptualizations of abusive men as “evil” or “narcissists” (Karageorgos et al., 2023). These ideas are frequently used either explicitly or implicitly in popular media, and as such represent one cultural resource boys might draw on to make sense of male violence and abuse within their family.

In contrast to these concepts of toxic or fragile masculinity, some theorists have explored what it might mean to be a “good man.” Beginning with Levant and Pollack's new psychology of men framework (Levant & Pollack, 1995) theorists and researchers have sought to make visible ways of doing and being men that do not play into patriarchal power and aggression. Other dominant contemporary representations of masculinities tend to be more inclusive (Blanchard et al., 2015; Fuller, 2023; Gough, 2022), and studies on boyhood in the last 10 years have documented how boys both recognize the connection of masculinity to violence and aggression, but are also able to find ways to define themselves as masculine *and* egalitarian and non-violent (Frosh et al., 2003; Fuller, 2023; Hamlall, 2013), emotionally expressive, and relationally tactile and flexible (Anderson & Mccormack, 2015; Way et al., 2014). The idea of “healthy or positive” masculinity emphasizes the value of “good men” who protect the weak (Sundaram, 2013), or “good fathers” who are strong but nurturing providers (Cole & Patterson, 2022). These constructs are deployed in violence prevention work, invoking men to “stand up” to abuse, to “protect women,” and be “real men”; such efforts however, have been criticized for entrenching the very patriarchal orthodoxy that prevention efforts must ultimately overturn (Dworkin et al., 2015; Pease, 2019; Seymour, 2018; Tomsen & Gadd, 2019).

If masculinity is, as Wetherell and Edley (2014) suggested, a set of *practices* that are negotiated in specific contexts, then the relational context of the family must be a key site for the working through of masculinities and femininities. When the context of the family is permeated with violence and control, it seems inevitable that this will impact the way that boys frame their own masculinities. In our article we explore how boys draw on and resist dominant notions of masculinity as they make sense of their relationships with their abusive fathers, extending Waling's (2019) point that men are not just victims of masculine ideologies, they have agency in their navigation of them.

## Method

“Understanding Agency and Resistance Strategies” was a 2-year research project, exploring children's capacity for agency and coping in situations of domestic abuse, in four European countries—the UK, Spain, Greece, and Italy. The interviews were conducted in 2014, and focused on how children coped with domestic abuse. This paper is based on a re-analysis of these data, focusing specifically on the experiences of boys who took part in the project, to consider what they said about their relationship with their fathers.

### Interviews and Creative Methods

We used semi-structured interviews (Eriksson & Näsman, 2012) and drawing-based approaches (Dumont, 2008; Gabb & Singh, 2015) to facilitate children's articulation of their experiences of relationships, and their ability to cope, to resist, and to maintain a sense of agency. As part of the semi-structured interview, children and young people were invited to draw pictures of their family, and maps of the home they lived in when the violence occurred. These were embedded in the interviews, using a “draw and tell” technique (Driessnack, 2006). Interview questions did not focus on the detail of the violence itself, instead exploring the ways children and young people found to cope while in a family where violence occurred, and in the aftermath. An interview schedule was developed to guide the interviews, and was used flexibly, to support children in narrating their experiences.

Most of the interviews were conducted by one researcher in each context, but there was a little variation cross-nationally, as inevitably there were departures and new staff joining over the course of the project. The interviewing team was trained together in semi-structured interviews, and we had ongoing conversations about how the interviews were progressing throughout the lifespan of the project. Interviews were conducted in the main official language in each national context (English, Castilian Spanish, Greek, and Italian).

### Participants

Within the four countries, over a period of 12 months, we conducted interviews with 107 children and young people aged 8–18 years, who had experienced domestic abuse but were living away from such situations or who were assessed by professionals to be safe to work with. Most participants came from families where the main perpetrator of the violence was identified as male. This article is based only on an analysis of the interviews completed with boys (44 in total). For this paper, we selected analysis from interviews where the father was clearly identified in children's accounts as the perpetrator of domestic abuse, and where boys talked explicitly about their relationship with their father. Thus, 31 interviews were included in the final analysis for this paper (see Table 1).

**Table 1.** Participant Information by Country.

Country	Number of boys who participated	Number of boys perpetrator is identified as their father	Age (range and mean)
UK (England)	9	7	9–15 years (10.1 years)
Greece	10	7	10–17 years (14.5 years)
Italy	18	11	9–18 years (14.15 years)
Spain	8	6	13–17 years (15.13 years)

Children and young people were recruited through domestic abuse services and social care services within participating countries. (Unfortunately, data on recruitment and service engagement were not consistently captured in each country and so are not reported in greater detail here.) Interviews were generally conducted in the context children were recruited from, in a safe, private room. This was facilitated by professionals working with families affected by domestic abuse, who made the details of the study available to potential participants. Families were directly approached by a member of the research team if they expressed an initial interest in the study.

### Analytic Process

The interviews were audio-recorded and subsequently transcribed verbatim. These were analyzed using reflexive thematic analysis (Braun & Clarke, 2006, 2021). Our analysis was guided by a constructionist ontology that viewed children's accounts and capacity for agency as socially, relationally, and materially constituted, rather than being an essential quality or production of the individual child (Raithelhuber, 2016). Thus, our analysis was inductive and understood as intersubjectively constituted (Byrne, 2022). We followed the steps suggested by Braun and Clarke (2006, 2021), familiarizing ourselves with the data through reading transcripts and listening to audio recordings, before using open coding working systematically through each transcript to identify aspects of the interviews that were relevant to the research questions. Interview transcripts were coded independently by two members of each research team, before each was shared and discussed to refine the coding strategy. We worked reflexively to construct the analysis, and to build consistent interpretive practice across the research team. Analysis was guided by a constant querying of how interpretations were rooted in the texts. Patterns of similarity and difference were explored within and between transcripts and country datasets, and from these interpretive themes were constructed.

### Reflexivity

Reflexivity was a central process in the study. Each individual researcher maintained reflexive notes on interviews immediately after they were completed, and these were read alongside transcripts of the interview. Throughout the analysis, researchers worked together to reflect on the coding and themes being constructed, exploring together why we were reading the transcripts as we did, and challenging each other to consider alternative interpretations and to elaborate our own positions in relation to the data and the study questions (Olmos-Vega et al., 2023).

### Ethics

The study was conducted in accordance with the ethics framework of the British Psychological Society (2009). In setting up this study, a key ethical concern involved navigating the tension between protecting participants from harm, and recognizing their capacity for agency, their right to self-expression, and their reflexive competence (Cater & Øverlien, 2014; Eriksson, 2010; Staf & Almqvist, 2015). We worked closely with relevant professionals to ensure that participants were in safe situations at the time of the interview, and that if children were distressed, there was support in place for them. Prior to gaining verbal and written consent, we ensured that all participants and their non-violent carers were fully informed of the purpose and focus of the interviews and of their rights to withdraw and omit questions. Consent^3^ was obtained from all children. If they were under 16, consent was also obtained from their non-violent parent or guardian. Anonymity was established through the use of pseudonyms, and the alteration or removal of any specific detail in the interview that might make the child or their family members identifiable.

## Analysis

Three superordinate themes were produced in the analysis of the data: (a) relational ambiguity; (b) performing masculinities, managing violence; and (c) envisioning alternative futures, re-visioning the past.

### Relational Ambiguity: The Good, the Bad, the Messy

Boys’ relationships with their fathers were typically ambiguous. Many of the boys saw their fathers as potentially dangerous, threatening, and at times manipulative. For example, speaking about his experience of being in refuge, Josh (UK, 9): “It felt a bit safe because he didn’t know where we were.” While Josh did not directly describe his father as dangerous, it is implied by the sense that Josh felt safer knowing his father did not know the family's location. For many of our participants, the father's presence was associated with fear and a sense of dread and danger. The choice of pronouns (“he” and “we”) suggests a symbolic separation of the father from others in the family; the father's exclusion from “we” supports a sense of collective safety. This is consistent with Lapierre et al.’s (2022) assertion that no or limited contact with fathers was a “relief” to children.

**Figure 1.**
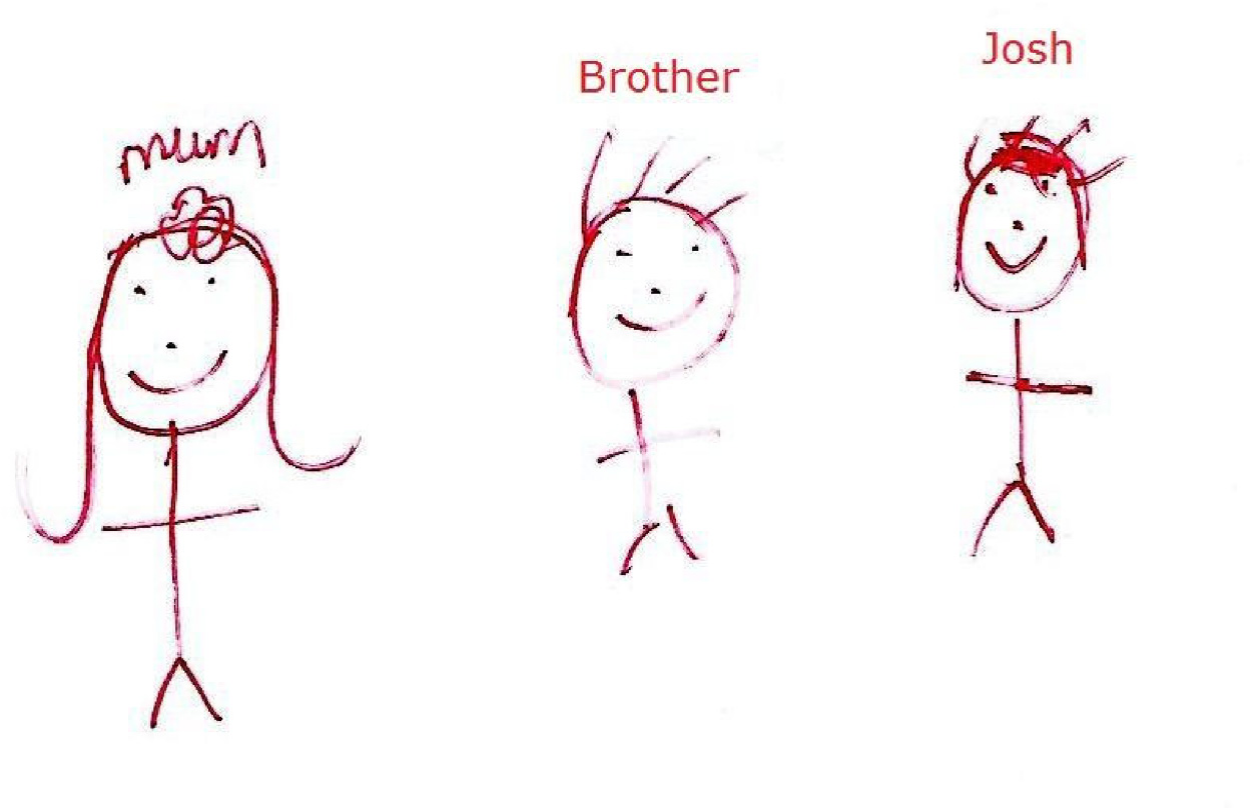
“I don’t want my mind to think that he's my dad,” Josh, family drawing.

Fathers often appeared to be absent in both children's narratives and their drawings. For example, where children were invited to complete family drawings, most of the children's drawings did not include a father, and in the two that did, fathers were described in negative terms while drawing (see Figure 1).

Many of the boys explicitly and deliberately excluded their fathers from their definition of family:

Int: …Josh, I notice you don’t call him “Dad.”

Josh (9, UK): No, I don’t call him “Dad”

Int: Did you ever call him “Dad”?

Josh: erm not really, I just called him Fred, even at home when I still lived with him

Int: Why was that?

Josh: Because I don’t want my mind to think he's my dad, ‘cause he's not, I don’t want a dad that hits me.

By refusing to call his father “dad,” Josh drew clear boundaries, explicitly and consciously, that excluded his father from the category of being a father. This is echoed by Elia (16, Italy):

Well … he is my father … he is a bastard … but I don’t care about him … he is happy when he stays with other people … and we feel better without him.

Here Elia suggested that while his father “is his father,” he was not someone with whom he had a positive emotional relationship. He implicitly excluded him from his sense of family (“we feel better without him”) and from any emotional involvement (“I don’t care about him”). “He” was not part of “we.” This exclusion of fathers from the definition of “family” seemed to be a conscious strategy for managing the emotional complexity of boys’ relationships with their fathers. These boys, in common with other interview participants, defined their father as something other than “father”—as just another man, as a “bastard,” as “not my dad.” On the one hand, they recognized a biological reality that this man was their father. But by making their own decisions about what that meant to them, about whether they accepted him relationally *as a father*, the boys could restore some sense of agency and control, as well as subjective safety.

**Figure 2.**
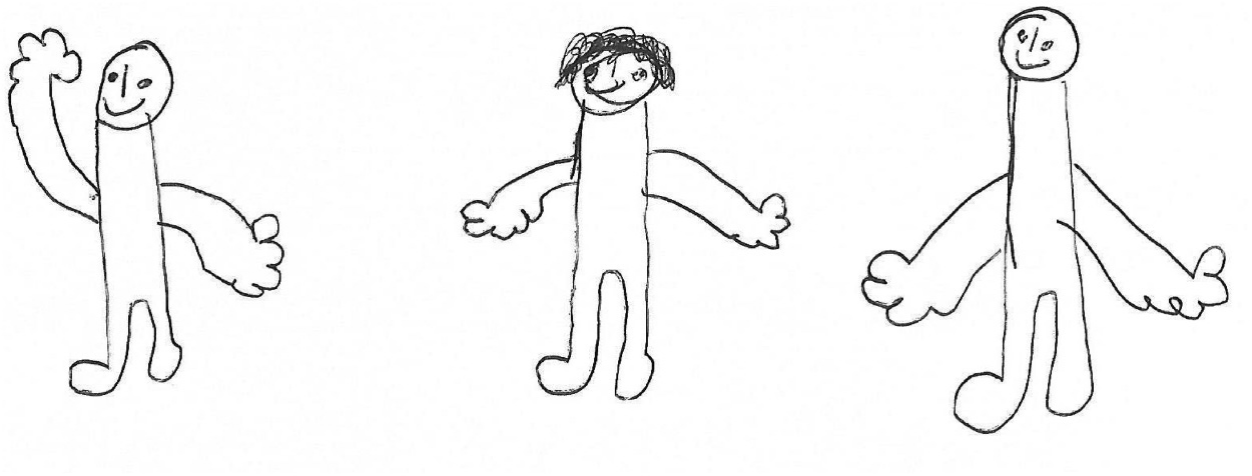
“The court said that we couldn’t see our dad anymore. I wasn’t really angry,” Oliver, family drawing.

This representation of the perpetrator as “not-father” was common in boys’ accounts. This is potentially an effective short-term strategy for dealing creatively with the challenges of living with a violent parent. However, the lived reality of their experience of their fathers was often more complex and ambivalent, as extensively discussed in a previous paper (Fellin et al., 2019). This ambivalence is illustrated in extracts from our interview with Oliver (UK, 12). He described his father as violent, suggested that contact with his father brings back bad memories of the way his father treated his mother, and stated that, for this reason, he did not want to have contact with him (Figure 2):

Like hitting and like calling my mum stuff ((.)) and I don’t like it when he says that so ((.)) it reminds me when I see him.

However, as the interview unfolded, Oliver's account of his father becomes less one-dimensional, more ambiguous, and more complex:

The court said that we couldn’t see our dad anymore. I wasn’t really angry ((.)) so I was okay, and we haven’t really seen him since… Well like not like spent time with him but I’ve recently, when I was walking back from school two, I think two times I’ve seen him, but that was just, like he was trying to talk to me, but I didn’t want to, but I didn’t really like spend the day with him or anything.

Here, Oliver explained that he continued to see his father as a threat, and that he did not want sustained contact. However, his phrasing also suggested traces of more ambivalent feelings about his father. For example, he positioned himself as accepting the court's decision that he would not have contact with his father, saying “I wasn’t *really* angry” (our emphasis). The interviewer had not asked if he was angry. In denying the feeling of anger, *he* seemed to introduce the feeling into the conversation—as if anger were perhaps something he did in fact feel, but could not easily state. Further, he qualifies the sense of anger (“I wasn’t *really* angry”), in a manner that suggested that perhaps he was more upset than he felt able to admit. This suggests greater complexity than is suggested in analyses like Lapierre et al.’s (2022), which emphasize children's purely negative attributions.

When asked about experiences of contact, Oliver (12, UK) said:

Oliver: Um it was kind of fun. Sometimes when he's like ((.)) like not really angry he like, sometimes we go out and we just have fun, and it's okay ((.)) but like I still keep in mind like what he he's done, so, but um so I kind of love him, but then I don’t because I keep thinking like what he's done…

Oliver seemed to hold two simultaneous images of the father, as a violent individual who has abused him and his mother, and as a person who can be fun, and who he (kind of) loves. “Remembering what he's done,” his father's capacity for violence, functions as a kind of talisman for Oliver, against the potential for his father to hurt him (and his mother) again. Loving his father was difficult for Oliver to admit (he “kind of loves him”), as if loving him were unacceptable or shameful, perhaps hinting at Oliver's unacknowledged sense of divided loyalties. The effect of the dichotomous representation of fathers as either “all bad” or “good” makes it difficult for him to resolve the competing images he holds of his father as both violent and problematic, *and* as someone he loves.

In this theme, we have highlighted the strategies boys use to manage their relationships with their perpetrator fathers. On the one hand (consistent with Lapierre et al., 2022), they used polarizing strategies, representing their fathers as violent, overwhelming, and negative; they symbolically evicted fathers from their construct of family. On the other hand, a subtler reading of their accounts suggested more ambivalence in these relationships than might originally be surmised. While the boys reject male strength and power performed through violence, they also recognize the complexity of their own feelings about their fathers, and their ambivalence about masculinity itself.

### Performing Masculinities, Managing Violence

While all young men must negotiate the complexities of masculinities as they develop and mature, this can be more challenging when masculinity intersects with other marginal social positions (Archer & Yamashita, 2003). The behavior and attitudes of domestic abusers are often “hypermasculine” (Archer & Yamashita, 2003; Ray & Parkhill, 2021), macho, and dominating. Consequently, for boys and young men growing up with domestic abuse, working through their feelings about masculinity and about themselves *as boys and men*, involves working with the ambivalence of their feelings about their fathers, and the caricature of masculinity that their behaviors embody.

Boys often described their fathers as irrational and unreasonable, with unregulated emotions:

Nacho (13, Spain): every time he came the door shook; he opened the door and he started to insult everybody…. When he was out of control, I knew him, he was crazy. He started shouting … saying: “I’m going to throw everything; I’m going to burn everything around…”

Nikolas (13, Spain): My father is hysterical, he doesn’t realize anything, he's always trying to be right, and he often gets angry.

While “good masculinity” is not typically associated with open expression of emotion, emotional control is generally seen as masculine (McAllister et al., 2019). Describing fathers as volatile, irrational, “crazy,” “hysteric” clearly positions them relationally as dangerous and unpredictable. Further, the language used by some boys to describe perpetrating fathers often draws on negative tropes of femininity—hysterical, crazy, manipulative—representing their perceived lack of emotional control as a kind of failed masculinity. In this way, the boys appear to resist the conflation of masculinity with violence, instead positioning lack of restraint as a non-masculine trait—good masculinity is framed as being able to regulate yourself and your emotions (Hamlall, 2013), as being both physically and emotionally strong and steady. This highlights a complexity in masculinity that boys need to navigate—the sense of being emotionally inexpressive, but also well-regulated.

Some of the boys we spoke to found relational ways to resolve the complexities of masculine identities, through the formation of “manly” but “caring” relationships with others in their families. These relationships enabled them to enact traditionally masculine roles like “protector,” without compromising their ability to express care and emotionality. Luca (17, Italy) expresses this in relation to his sense of his obligations as “the man” in his family:

With my brothers I feel compelled to protect them because they are small, they no longer have a male figure… I feel obliged to help them with everything, from study to small everyday difficulties… Sometimes I and my brothers were hiding in the room. To stop them from getting scared too, I pretended to play, to play hide and seek… I would try to comfort my brothers.

He takes on the traditionally masculine role protector, enabling his brothers to find a safe space within which they could maintain some semblance of “normal childhood.” He describes himself as providing comfort to his brother, of playing with them to stop them from feeling scared. While on the one hand, he felt that caring, even *fathering* was required of him, this caring role also offered him various positives too: he is able to be the substitute father figure, the Big Brother, able to care and feel competent in his protection of his brothers (a powerful self-positioning) and he is also able to find space to play (space to be a child), even as the conflict goes on. The possibility of building a positive masculine identity in the caring role is clear as Luca relates to this more as protection than care.

Being “the man of the house” was a common preoccupation for the boys we interviewed, particularly the ones who were older brothers. Being “the man” involved taking on responsibility and duties that, in many senses, they felt that their fathers had not taken on:

Angelo (15, Italy): I feel I have to protect my brother because he is younger than me and he does not have a paternal figure. I feel I have to help him with homework or when he quarrels with someone. I like to go to speak to his teachers with my mom.

Elia (16, Italy): (I’m close to) both my mother and my sister, equally: I’m the man of the house, I have to look after them. Maybe cos in my blood… I mean in my veins … runs Southern blood… I feel I’m the man responsible for the family.

Like Luca, Angelo experienced stepping into the role of father as a moral obligation. He takes on a parental role, supports his brother, helps him with his homework, and stands alongside his mother as a kind of quasi-adult/father. Being the “man of the house” seems to confer on the boys a strong and positive masculine identity, which they feel they are positioned to assume when their father is excluded from family life. They fill the masculine space their father has vacated; in several cases, they were the ones who encouraged their mothers to flee and/or those who first sought help from police or other professionals in order to end the abuse. This might be problematized as an expression of parentification, it does also serve a potentially positive and transformative function (see also Callaghan et al., 2016). In “fathering” their siblings, and allying with the mother, the boys are engaged in a kind of reparative care giving, “fixing” their own relational difficulties with their fathers by accepting a paternal role, and drawing together the elements of protection and care that combine in dominant representations of what a good father is. Rather than perpetuate violence, they appear to resist the father as a role model in favor of a more caring, emotionally resilient version of masculinity. They resist the practices of hegemonic masculinity (Connell & Messerschmidt, 2005), forging for themselves a masculinity that is strong, but kind, caring, and emotionally responsive.

For some boys, this sense of masculinity as *protector* extended into physical attempts to protect their family and resist their father's violence:

Nacho, (13, Spain): As I was beside her (his mother)… I tried to order him to stop, I lifted her and I told him to stop… But he brought me and bump!!

Mark, (13, UK): He went, because I told him to get out of the house, or I’d hit him again and he got out of the house, and I didn’t see him then…. And my mum wanted him out as well, so I said, “Get out.”

In both these extracts, the boys assert their masculine positions as protectors and defenders of the family, resisting their fathers’ problematic and violent performance of aggressive masculinity and responding with a more protective form. Both boys—but particularly Mark—are drawing on the “man of the house” discursive construction to resist the father's violence. Mark aligns himself explicitly with his mother, asserting her desire that he “get out.” Paradoxically, Mark takes control of the space through his own use of violence and his assertion of masculinity. A similar construction is used by Franci (15, Italy):

Before, I was a child. Now I can defend myself and my family better. I practise kickboxing; I can hurt him now.

Here Franci represents his prior helplessness as a childish trait—he was unable to defend himself and his family. He contrasts this with a new position—the term “now” suggesting a more adult, manly sense of self. But, positioning himself as a man, he feels more able to offer an embodied resistance to his father's violence. The sport he practises gives him a better sense of control and of an ability to protect himself and his family. He is able to manage his body aggressively, defensively, but in a controlled manner, through the discipline of his sport. “Growing up to be a man” in his family means on the one hand taking on the hegemonic masculine role of protector, and on the other hand, being able to be *competently and healthily* violent. Being a man means being aggressive—even if that aggression is channeled into protection rather than aggression. The boys are drawing on the acceptable construction of *controlled* male violence as protecting the weak (Cole & Patterson, 2022; Sundaram, 2013). This enables their positioning as “good men,” but does not challenge the production of this construct within dominant constructions of hegemonic masculinity (Tomsen & Gadd, 2019).

For many boys, this experience of becoming a violent protector, of taking on masculine aggression, does not sit entirely comfortably. While being a protector and the man of the house may superficially seem a source of pride, it is not straightforwardly positive. Their stories of retaliation and protective/defensive violence is often told in ways that hedge the adoption of that violence, positioning it as something that did not “fit” with their usual behaviors, or that was somehow *not* them. Consider this example. Mark (13, UK) describes a scenario in which he engaged in retaliatory violence toward his father:

Mark: He threatened my mum, saying that if you come near me, if you say—if you like wind her up again apparently, if she winds him up again apparently that's what she did, he’ll throw a door at her. So I went mad at him.

Int: Ah, so were you protecting your mum?

Mark: Yeah. … I had to.

Int: You had to, why?

Mark: Because my knee hurt and my sisters were crying, I just went mad.

Int: Was that the point that you hit him?

Mark: Yeah.

Mark, as a 13-year-old child, hit his father; indeed, he hit him hard enough that he was questioned by the police about his actions. In this extract, Mark presented his actions as a more acceptable expression of male violence, protecting his mother and sister, and defending himself. Mark's account of this act of retaliatory violence is characterized by a sense of compulsion (“I had to”) and justification (“Because my knee hurt,” “because he threatened my mother,” “because my sisters were crying”). It is clear in the extract that the violence is something he felt uncomfortable about: he felt out of control (“I just went mad”) and was frightened by his own physical response to the threat of his father. Positioning it as something that was forced upon him by circumstances, and that was beyond his control, and for protecting female victims (mother and sisters), helps defend against any possible accusations that he himself has become violent. He clearly positions violence as something he did not want to do, something he was forced into, but the anxiety about becoming what he beheld is still clearly in evidence in the subtext of the quote, and the justificatory frameworks he put around the action (perhaps also as a reaction to the emphasis on intergenerational transmission in evidence in services that he has used). This ties into the concerns many boys express about reproducing masculine violence, and becoming *just like their dads*.

In this theme, we have explored how boys talk about and navigate their self-positioning, as boys and future men, navigating the violent and problematic masculinity represented by their fathers. We examined how they drew on alternative representations of masculinity—as strong and caring, as protector—to construct a more positive self-representation. However, the ambivalence of this positioning is still clear; there are concerns about being aggressive or violent in their protectiveness, and still draw on ideas about being “manly men.” The protector/carer role is therefore not an unambiguously positive positioning, and the boys’ own ambivalence is visible, particularly in their sense of *obligation* to be a substitute parent. Nonetheless, this positioning does suggest a potential positive space for engagement with constructions of masculinity, from which a more flexible way of being a man and a father might be negotiated.

### Envisioning Alternative Futures, Re-Visioning the Past: Managing Anxieties About Intergenerational Transmission

The young boys in this study expressed (directly, and indirectly) anxiety about being “like” the perpetrator, and fears about taking on the violent parent's aggression, relational challenges, and irrationality. Given the popularity of the discourse of intergenerational transmission in professional work with families affected by domestic violence and abuse (Black et al., 2010), this anxiety about learning the perpetrator's behaviors is often entrenched by well-meaning professionals (Callaghan & Alexander, 2015). This is further underscored by a service model that, when it does concern itself with children, tends to emphasize managing their problem *behaviors*, a model that is dependent on a view of children as passive recipients of environmental learning. The fairly common practice of not housing older boys in women-only refuges also inadvertently produces a representation of boys’ bodies, of masculinity itself, as inherently dangerous:

Niko (15, Greece): Ok, then we had gone, in the beginning ((.)) to the nuns… To stay all together, and because big chil-, big boys are not allowed… I had gone when I was eight years old. I had gone to another that, to another institution/orphanage.

He begins to suggest that big children are not allowed in refuge, but corrects himself to *big boys*, demonstrating an awareness that it is masculinity itself that is problematized in the exclusion of boys from these organizations. This practice clearly communicates to boys an implicit assumption that boys and men are necessarily potentially dangerous to women and girls.

A concern about repeating their fathers’ violence is a common concern among boys who experience domestic violence and abuse. It also indicates a conscious effort to make sense of their own feelings and behaviors, and by making sense, to take ownership of them. In this way, Alberto draws on an understanding of his father's personal history, using an explanation for his violence that is rooted in the notion of intergenerational transmission to make sense of the way that his father became violent:

Alberto (15, Spain): Yes, but I think he was treated like that when he was a child. I think it is not his fault because he had the same situation of violence when he was little. So, I cannot expect something different from this kind of person, but I don’t want him to hurt me.

Alberto uses his insight into his father's history of violence to make sense of his behavior, almost absolving him of blame. However, he also sets appropriate boundaries around that understanding, expressing firmly that while his father's experiences help him to *understand* the violence, they do not *justify* that violence or remove the hurt that accompanies it. This insight functions to interrupt the intergenerational transmission process for Alberto. He sees the cycle as predictable, understandable, and sees his father as repeating that cycle relatively choicelessly (“I cannot expect something different from this kind of person”). Nonetheless, he does not fully absolve his father of responsibility. However understandable his father's violence might seem, nonetheless Alberto does not want to be the victim of such violence (“I don’t want him to hurt me”).

Despite the pervasive discourse of intergenerational transmission, which positions boys’ perpetration of family violence as more likely for boys than for girls (Stith et al., 2000), boys we interviewed were nonetheless able to envision alternative ways of being, for themselves and for their fathers. For example, Petros (16, Greece) says:

Petros: The more I was growing older, the more I was comfortable to say, to talk to my father … “why are you fighting? Don’t do this… There's no need to fight in front of us”. … to stop him, to explain to him that what he is doing is not right. ((.)) then I had a little success. Through what I was trying to do… Every time I was having a conversation with him, I understood, I knew more things than when I was younger. And I could understand and ((.)) give him some advice.

With maturity, Petros was able to understand and contextualize the violence, and to make meaning of his father's actions. This did not mean that he accepted his father's violence, but he was able to give it a meaning in the context of his own life, and to talk to his father about possible alternative ways to behave. Here, Petros chose to command respect through talk, not violence. While other boys felt they needed to take on *strong protector roles* to enact an alternative possible way of doing masculinity, Petros suggests that reasoning with his father, helping him to see different ways of behaving, and offering him advice was a possible strategy to resist his father's violence, and to build an alternative masculinity for himself. This is a different way of growing up to be a man. Maturity enables him to use reason and calm logic, to work with his history and with present violence.

To envision a violence-free future for themselves did require that they find a way to make sense of their father's violence. For some, this was about their fathers’ own histories of violence and abuse, for others, it was understanding the way that rigid hegemonic masculinities might function to entrap men in violence. For instance, Elia says:

But these difficulties have been training for the challenges of life and so, now, I’m not afraid of anything. When I was a child, I always had to move house, school and friends. And we were not fine. My father—maybe he wrote himself off cos he didn’t become a famous (football) player. He only played some matches in an important championship, but he was a reserve. He was angry, and he yelled out at us.

He makes sense of his father's anger and violence as an expression of failure as a man—his failure to live up to the masculine ideal of being a famous footballer. Elia draws on a stereotype of masculinity—the idea that men's violence and rage is often an expression of threatened masculinity (Cohn et al., 2009)—as a resource to make sense of his father's behavior. Having failed at football, Elia suggests his father's violence was a kind of impotent rage, that he took out on others. Elia shows understanding of his father's disappointments and his inability to appropriately express his frustrations; however, Elia sees himself as transcending that history. He suggests it has made him unafraid, able to face down adversity—in many senses what he might define as “a true man,” as a foil to his father's *failed* masculinity. In reframing their fathers’ rage and violence as failed masculinity (as disappointment, failure to perform, failure to regulate emotion) the boys are able to rehabilitate the construct of masculinity for themselves. By locating the fault with the perpetrators’ *failure to be masculine*, they open up a space in which they can be men, but not *this sort* of man.

Many of the boys we spoke to were able to articulate alternative positive futures for themselves, re-working their histories in a way that released them from the sense of being doomed to repeat their fathers’ violence. Santo challenges the idea that he is doomed to be a bad father, asserting his belief in his potential to parent well:

Santo, (18, Italy): I do not know. In my case it was not so. But I feel that one day I could be a good father, because I learned how to manage my situation and I want to improve myself even more. I do not intend to repeat my family's mistakes when I have a family too.

He sees himself as learning from familial mistakes, because he has achieved some insight into what they are, and has developed a better understanding of how to cope with his feelings and reactions. The boys re-interpret and re-vision their histories of violence as learning environments, in which they have acquired insights into how *not* to be a father, and position themselves as transcending those histories through the personal strength they have acquired as a consequence of the problems they have experienced. This gives them a sense of hope, of positive aspiration, beautifully articulated by George:

George (11, UK): But if I had to plan my life, I’d have education, then a house, then a car, and then children….

Int: When you grow up, what kind of parent do you think you’ll be to your children?

George: Really nice. Yeah. ‘Cause what happens, most of the dads are nice. So, it keeps going on nicely.

Here, George lays out for himself a future in which he is just a dad, like “most of the dads.” His future self has an ordinary education, an ordinary job, a home, and children. And like “most of the dads,” “keeps on going nicely.” George explicitly resists the positioning of himself as inevitably violent, recognizing that violence is not “normal” for most men and boys, and that being a *nice man* and a *nice dad* is a perfectly reasonable aspiration for himself.

In this theme, we have considered how boys have deployed visions of their future selves as a kind of symbolic antidote to their anxieties about becoming like their fathers.

## Discussion

Boys who experience domestic violence and abuse find themselves in a complex position, having often to forge an identity for themselves as boys, becoming men, in an environment where a key influence on their lives is a man who is violent, dominating, and controlling. In contrast to the relative passivity predicted by the model of “intergenerational transmission,” the boys we interviewed navigated competing constructions of the positions of victim and aggressor, and of masculine and feminine, to negotiate social scripts about what they *should be* as they grow up to be boys and men.

Boys who experience domestic violence and abuse often have ambivalent relationships with their fathers, as they attempt to negotiate very dichotomous representations of men and masculinity. On the one hand, they view their fathers with fear, distrust, and sometimes dislike. The image of fathers as dangerous that we have documented here is consistent with the findings of other qualitative studies (Cater, 2007; Lapierre et al., 2022; Øverlien, 2014; Staf & Almqvist, 2015). Their threatening and coercive behavior is seen as frightening and as challenging to other relationships (Callaghan et al., 2016). On the other hand, in line with Henze-Pedersen's ethnographic study (2021), many of the boys we spoke to still had positive memories, and in some cases desired a relationship with their father moving forward. In a discursive milieu in which abusive men are positioned as violent objects, it is difficult for boys to negotiate these different experiences of their fathers. There are few resources available to support boys in understanding their feelings about fathers who are *both* violent and challenging, and men with whom they have had (and/or could still have), at times positive, relationships (Callaghan et al., 2019; Fellin et al., 2019).

Symbolically blocking out or excluding the father from notions of family in this way is a creative and (in the short term) effective inward-looking strategy (Mullender et al., 2002), but can also produce challenges for children. The cognitive strategy of excluding the father from the definition of family does not necessarily indicate a successful emotional exclusion of him, nor does it enable the integration of the experience of family violence into their own or their family-narrative (Cooper & Vetere, 2008). It prevents the emergence of a realistic, integrated image of the father and of masculinity that acknowledges and encompasses positive relational elements (however limited and conflict-laden they may be) as well as the negative. Paradoxically, this strategy may trigger a greater level of identification with the excluded abuser (Vetere & Cooper, 2017). This dichotomous representation reproduces familial and service context representations of abusive fathers as relatively dehumanized, reduced to a mere violent object, and not a relational subject, potentially positioning the fathers (and by extension maleness) as irretrievably damaged, and as a consequence, their sons as doomed to the cycle of violence too. Thus, silencing experiences of ambiguity, constraining their articulation, and polarizing mothers and fathers, make it challenging for boys to develop an integrated sense of self that acknowledges their lineage and familial roots, and that can function as a basis for a different, non-violent identity. It is important to note, this is not a recommendation for boys to have continuing contact with and relationships with their fathers, particularly where contact poses safety concerns. Rather it is an appeal to construct spaces in which fathers are not unspeakable for children, in which they can work through the complexities of these relationships.

As Cooper and Vetere (2017) remind us, open conversations about the relational meaning and effects of domestic violence and abuse, blame, and shame can help to change and deconstruct narrow and unhelpful definitions of masculinity and of family relations that are often transmitted over generations. Enabling children and their parents to talk openly about relationships and the impact of violence is crucial to preventing their experiences from becoming “shameful secrets,” “frozen,” and inarticulable (Cooper & Vetere, 2008, p. 70). Creating space for boys to talk about their fathers, and their relationship with their fathers is therefore important to enable boys to understand their fathers’ behavior and to build a sense of a different masculinity for themselves.

By understanding masculinities as a set of *practices* that are negotiated in specific contexts (Wetherell & Edley, 2014), we have been able to consider how the relational context of the family, and particularly the emotional and relational spaces between boys and their fathers, function as a key site for boys to navigate their positioning as *masculine.* Most research on boys’ relationships focuses on the way masculine roles are rigidly policed, particularly in relation to close relationships, where shaming and aggression are used to discipline masculine expression and produce limited spaces for emotional expression in boys’ relationships (Stoudt, 2006). The open expression of vulnerability, hurt, intimacy, and emotionality is largely disallowed within hegemonic constructions of masculinity (Bartholomaeus, 2012). In our interviews, boys’ ability to flexibly use the concept of the “man of the house” to express closeness and care shows that masculinities do not straitjacket boys emotionally (Connell & Messerschmidt, 2005), and that boys value the positive personal consequences of sharing emotions, and caring for others, particularly in times of distress (Oransky & Marecek, 2009). In this sense, boys are performing a very complex kind of masculinity work.

Boys are able to re-envision both their relationships with their fathers, and their understanding of masculinities and themselves as masculine, to create spaces in which they can view themselves as men (or becoming-men) *and* non-violent, and as caring young men. This requires a realistic apprehension of their father as a full and flawed human being, capable of horrible acts of coercion and violence, but also someone who they sometimes loved and whose company they sometimes enjoyed. In envisioning a positive alternative future for themselves, the boys we interviewed resist a dichotomous representation of themselves as either violent, dominating aggressor, or weak helpless victim. We argue that these positive visions of the self offer a potential foundation for boys to build alternative visions of themselves.

### Limitations

The data for this article are drawn from a larger study of children's experiences of domestic abuse, that was not specifically focused on father–son relationships. The interviews reported here were conducted in 2014. The second phase of this project (2015–2016) involved a therapeutic intervention with a different group of children, based on the interviews conducted in the first phase. In conducting the therapeutic intervention and its evaluation (Callaghan et al., 2019; Fellin et al., 2019), we learnt how central and concerning issues of masculinity and fatherhood were for the young boys in the therapeutic groups, where concerns about intergenerational patterns of abuse were also frequent themes. This was not a theme built into the interventions, but emerged as an agenda that preoccupied the young people themselves. For this reason, we returned to the interviews conducted as part of the first phase of the research, and re-analyzed the material relating to masculinities and fatherhood. Although these data are now nearly 10 years old, the issues described remain current and pressing, as responses to boys who have experienced domestic violence and abuse (DVA) remain underdeveloped (Perez-Martinez et al., 2023). These data were re-analyzed for this article, focusing specifically on sections of the interviews where children described their relationships with their fathers, or the impact of those relationships on them. Had this been a primary dataset focused on father–son relationships, it is possible that more complex accounts could have been provided. However, the father–son relationship was a dominant theme within these interviews on coping, and therefore the analysis here is justified, since it was an important topic for the young people themselves, and has continued to be a theme in subsequent iterations of the intervention we developed. The examination of fathers in the shaping, regulation, and socialization of children's emotions is also still very limited (Cherry & Gerstein, 2021), and research on intergenerational processes still tends to focus on questions of mere repetition, and not the meaning-making that young people might engage with around this process. The findings we present here have the potential to inform further therapeutic work that address these core identity issues in specific intervention programs for young boys who are victims/survivors of DVA.

This article provides snapshots of extremely complex dyadic father–son relations, but it is important to recognize that these are embedded in multiple systems of other relations, which contribute to children's understanding of relationships and of masculinities (Dallos & Vetere, 2012). In this article, we have not explored these broader relational networks, in order to redress the relative absence of children's accounts of their relationships with their fathers in the interviews. However, our focus on the father–son dyad does risk obscuring how this interconnects with other relationships and networks. It is not our intention to suggest that father–son relationships determine boys’ masculinities, or that they can be understood in isolation from networks of other relationships and contexts (Schrock & Padavic, 2007). More research is needed that explores the relationship between boys and their fathers, and that understands their relational experiences within their broader familial and systemic contexts.

Conducting research across a large-scale multi-national team has its own inherent challenges. Although measures were taken to stabilize the way we recruited participants, the way we interviewed and the way we analyzed data, nonetheless variations in local practice were identified as the project progressed. For example, there was variable practice in the recording of data on post-separation contact with perpetrators, and how reflexive summaries were generated that had some impact on how we were able to analyze data. Varying recruitment practices also may have impacted the data—for instance, in Greece, a number of the children were recruited through children's homes and these participants had quite different experiences from those who were recruited from within domestic abuse shelters in Greece and in other national contexts. In addition, it was difficult to track the varying service experiences of participants—for instance, the therapeutic provision they might had previously received was scattered and difficult to map due to lack of information sharing, gatekeeping, and other difficulties in referral patterns.

Different legal contexts made progress more challenging in some countries—for example, in Italy and Greece the requirement that perpetrator fathers consent to child participation if they shared custody had significant implications, also for recruitment. In each of the participating countries, our team worked closely with local stakeholders to adapt our work to specific and diverse populations. Nonetheless, there remains a need for future qualitative research to examine more explicitly the role of cultural and socio-economic conditions in the construction of masculinity and its implications for boys and men who experienced domestic violence and abuse.

## Conclusions

We have highlighted the need to challenge theoretical and practice models that set up self-fulfilling prophecies for boys, or that predict that children who grow up with domestic violence and abuse are likely to repeat cycles of victimization and perpetration. These models are fundamentally unhelpful in promoting children's recovery from domestic violence and abuse, particularly for boys who feel significant anxiety about repeating the violence of perpetrator fathers. Many boys who grow up in violent households do not become perpetrators. Instead, theoretical accounts and therapeutic work should facilitate the expression of boys’ ambivalent feelings and fears about their father's violence, and what it means for them and their future (Callaghan et al., 2018). This requires a more complex understanding of perpetrator fathers, and children's relationships with them contextualized in the family history, dynamics, and belief system (Cooper & Vetere, 2008).

In our experience group interventions that offer a safe place to identify, discuss, and value their own and others’ emotional responses and coping strategies, as well as difficulties in developing their own masculinity are particularly recommended (Callaghan et al., 2019; Fellin et al., 2019). This can offer boys a unique space for: (a) reciprocal sharing and better understanding and acknowledging of the complex relational dynamics and divided loyalties associated with the family violence (including positive aspects of fathers and ambivalence toward non-perpetrator parent and other family members) and the ambivalent emotions attached to them; (b) enhancing their sense of agency and of *positive masculinity*, by differentiating their developing self from that of their fathers’ and contextualizing violence in their family history; and hence (c) de-constructing self-fulfilling prophecies about intergenerational transmission and envisioning alternative futures for themselves as emotionally competent, strong, and caring young men.

Given the above-mentioned challenges and that these embodied and emotional experiences are very difficult to articulate, boys may need extra support in acknowledging and normalizing the complexity and ambivalence of their relational and emotional experience; to this purpose, creative activities and embodied techniques should be offered as alternatives or complement to verbal therapy.

Our findings also support the need for preventative interventions adopting a gender transformative approach in educational interventions to engage young people in critical thinking about hegemonic masculinity and to prevent domestic violence and abuse and interpersonal violence, including bullying and dating violence (Pérez-Martínez et al., 2023; Stanley et al., 2016; Yount et al., 2017).

Pérez-Martínez et al. (2023) found that machismo and acceptance of violence are widely present among adolescents in various countries but can be effectively tackled with these transformative interventions.

## Notes

1. The term “domestic abuse” is used throughout this paper, to refer to controlling, coercive, threatening, degrading, and physically violent behavior in the context of an intimate relationship or previously intimate relationship.

2. When discussing abusive male partners, we refer to “fathers and stepfathers” when talking generically about this role. When we specifically refer to fathers or to stepfathers singularly (not fathers and stepfathers) this is because this is accurate for the individual circumstances referenced.

3. We use the term “consent” not “assent” to refer to children's agreement to participate, since this better reflects our commitment to recognize the full personhood and agency of children and young people.
